# RFID Backscatter Based Sport Motion Sensing Using ECOC-Based SVM

**DOI:** 10.3390/s23177324

**Published:** 2023-08-22

**Authors:** Lei Han, Xia Hua

**Affiliations:** Department of Physical Education, China University of Petroleum (East China), Qingdao 266580, China; 20140034@upc.edu.cn

**Keywords:** radio frequency identification, backscatter, motion sensing, support vector machine

## Abstract

With the advent of the 5G era, radio frequency identification (RFID) has been widely applied in various fields as one of the key technologies for the Internet of Things (IoT) to realize the Internet of Everything (IoE). In recent years, RFID-based motion sensing has emerged as an important research area with great potential for development. In this paper, an RFID backscatter sport motion sensing scheme is proposed, which effectively solves the multi-classification problem by using the received signal strength (RSS) of the backscattered RFID and the error correcting output coding (ECOC)-based support vector machine (SVM). We conduct extensive experiments to validate the effectiveness of the proposed scheme, in which the signal intensities of different types of action poses are collected and the SVM is used as the classification algorithm to achieve high classification accuracies.

## 1. Introduction

With the thriving commercialization of the 5G network, the development of the Internet of Things (IoT) has been greatly facilitated by the high data transmission rate and low network latency of that network [1]. The essential link for IoT to actualize the Internet of Everything (IoE) is information-sensing technology. Currently, existing technologies such as radio frequency identification (RFID) [2], infrared technology, geomagnetic sensing, and video identification, are capable of connecting things to the internet in the form of information. Compared with other technologies, RFID technology has obvious advantages in terms of sensing accuracy, sensing distance, and application cost. Therefore, RFID technology, which achieves target recognition through signals between the reader and tags, has been widely applied in various fields [3,4,5,6,7,8].

Motion sensing has long been a popular and challenging area of research. We can learn or forecast crucial information by recognizing human postures, and then respond appropriately based on the results of the recognition. The use cases for motion sensing are quite diverse, with promising future developments in the areas of security monitoring, human–computer interaction, healthcare, game amusement, and the creation of film and television dramas.

Recent investigations have brought to light the expanding utility of RFID backscatter signals, transcending their conventional application domains and branching into areas like motion sensing [9,10,11,12]. This evolution paves the way for the exploration of RFID backscatter signals as a means to capture and discern human action poses, a realm teeming with potential advancements. Furthermore, addressing the intricacies of multi-class scenarios often entails resorting to the error correcting output coding (ECOC) strategy. This widely employed approach dissects the original multi-class conundrum into a series of binary sub-problems, rendering it a valuable tool in the arsenal for dealing with complex categorization tasks. In light of these considerations, this paper endeavors to resolve the multi-classification puzzle intrinsic to sport motion sensing. We present the ECOC-based support vector machine (SVM), harnessing the distinctive characteristics of backscattered RFID signals. Specially, we utilize the received signal strength (RSS) of RFID backscatter signals to measure the feedback of the sport motion. To address the formidable challenge of multi-classification in sport motions, we put forth a novel solution—the ECOC-based SVM scheme. This strategic approach takes advantage of the ECOC framework’s prowess in handling complex multi-class scenarios, offering a robust solution to a persistent problem.

The main contributions of this paper are summarized as follows:

(1) We apply the received signal strength (RSS) of RFID backscatter signals to sport motion sensing.

(2) We propose the ECOC-based SVM scheme to solve the multi-classification problem in sport motions.

(3) We conduct extensive experiments to validate the effectiveness of the proposed sport motion classification scheme.

The rest of the paper is organized as follows. Section 2 summarizes the related work. Section 3 presents the preliminaries to this paper. Section 4 presents a detailed motion-sensing system based on RFID tags. In Section 5, the proposed approach is evaluated and the results are discussed. Finally, Section 6 concludes this paper.

## 2. Related Work

Motion sensing, which involves learning or predicting important information by sensing the motion of the human body and taking prompt action based on perception, has attracted a lot of attention in recent years. Motion sensing has a wide range of applications and has been effectively implemented in many fields. In this section, we review the relevant research work in the field of human motion sensing.

The pose recognition algorithms mainly include depth map-based algorithms and RGB image-based algorithms. Yang [13] proposed the depth action map, which is obtained by accumulating the difference between adjacent frames of the depth sequence. However, the traditional depth action map projects the whole video sequence onto a single map, resulting in a significant loss of temporal information. Later, Xu et al. [14] divided the depth sequences into three parts to obtain their depth motion maps (DMMs) and depth static maps (DSMs), and then found their histograms of oriented gradient (HOG) features for each DMM and DSM. Although the recognition rate of this algorithm has been greatly improved, the computational complexity has increased and the real-time performance is not achievable. Oreifej et al. [15] proposed a new way of feature extraction in which the normal vectors of the target object are computed in four dimensions (including the time dimension) and histograms are used to obtain the distribution of these vectors. This algorithm produced excellent experimental results. However, these algorithms are limited by the functionality of the image acquisition device. Schuldt et al. [16] utilized spatio-temporal interest points to extract the feature expressions of RGB action sequences and used an SVM classifier to obtain the final classification results. In addition, Dollar et al. [17] proposed a method based on a sparse interest point to improve the algorithm.

The current application of sensors to recognize the human pose is also an important research direction in human motion sensing. Gu et al. [18] proposed an online activity sensing system that explores WiFi ambient signals to obtain the received signal strength indicator (RSSI) fingerprints of different activities, which can be integrated into any existing WLAN networks without additional hardware support. Huang et al. [19] proposed a device-free motion recognition method that exploits RF signals, such as RSSI, in a non-visual environment to extract features from received packets. This method is able to classify different motion directions and speeds, in addition to distinguishing human height based on the frequency characteristics of received packets. The WiQ system proposed by Lv et al. [20] is based on a deep neural network learning engine that extracts quality information from changes in signal strength, which can achieve high-precision sensing of the driving behavior. Using, for example, devices with Intel 5300 NIC capabilities, various changes in channel state information (CSI) can be generated through different human action poses. In addition, the technique of recognizing action poses based on the sensing of CSI signal state information achieves the sensing of corresponding action poses. Chang et al. [21] converted the received CSI into images by observing the similarity between channel state information and texture. They used a vision-based approach to extract features and train an SVM classifier for action recognition. Wang et al. [22] proposed a CSI-based human activity sensing and monitoring system, CARM, to quantitatively establish the correlation between CSI value dynamics and specific human activities. CARM uses this correlation as an analysis mechanism and recognizes a given activity by matching it with the most suitable profile. In order to intelligently estimate the driving behavior of commercial WiFi devices, Duan et al. [23] applied CSI amplitude variation data and proposed a scheme based on a back propagation (BP) neural network algorithm. The scheme filters sensitive input data from the raw CSI matrix of WiFi signals and achieves the classification of continuous driving activities by introducing posture sequences.

## 3. Preliminaries

In this study, a SVM is employed as a classification framework for human sport motion sensing, predicated on prior research involving RFID tags [2,3,4,5,6,7,8]. The SVM methodology derives its underpinnings from the Vapnik–Chervonenkis (VC) dimensionality theory and the structural minimization principle inherent in statistical theory. The approach is rooted in identifying the optimal trade-off between model complexity—reflective of the learning accuracy achieved with a specific training dataset—and learning capacity, which signifies the aptitude to discern diverse samples without incurring errors. This process operates within the constraints of a finite sample dataset, thereby striving to achieve optimal generalization performance.

### 3.1. Theoretical Basis

VC dimension [24] is a simple measure of a function as well as a description of the complexity of the problem. If the VC dimension is higher, then the considered problem is more complex. The structural risk minimization (SRM) [25] is to reduce the VC dimension of the learning machine while guaranteeing the classification accuracy (empirical risk), which allows the learning machine to control the expected risk over the entire sample set. Empirical risk minimization is commonly used in traditional machine learning algorithms. However, sometimes the obsessive pursuit of small training errors can lead to a reduced generalization ability and cause overfitting problems. The principle of SRM is to minimize the sum of empirical risk and confidence risk.

The equation for the generalization error bound is:

(1) $$R(w)\;\leq {\;R}_{\mathrm{emp}}(w)+\phi (n/h),$$

where R(w) denotes the true risk, $R_{\mathrm{emp}}(w)$ denotes the empirical risk, and ϕ(n/h) represents the confidence risk. Consequently, the inherent objective of the problem undergoes a transformation, shifting its focus from the singular pursuit of minimizing empirical risk to a dual pursuit that encompasses both the minimum empirical risk and the confidence risk, resulting in the minimization of the structural risk. This shift in perspective underpins the foundation of support vector machines (SVMs). By incorporating this paradigm, SVMs are constructed on the bedrock of balancing empirical and confidence risks, thereby attaining an equilibrium that fosters robust generalization and enhanced model performance.

### 3.2. Linear Classifier

**Figure 1:**
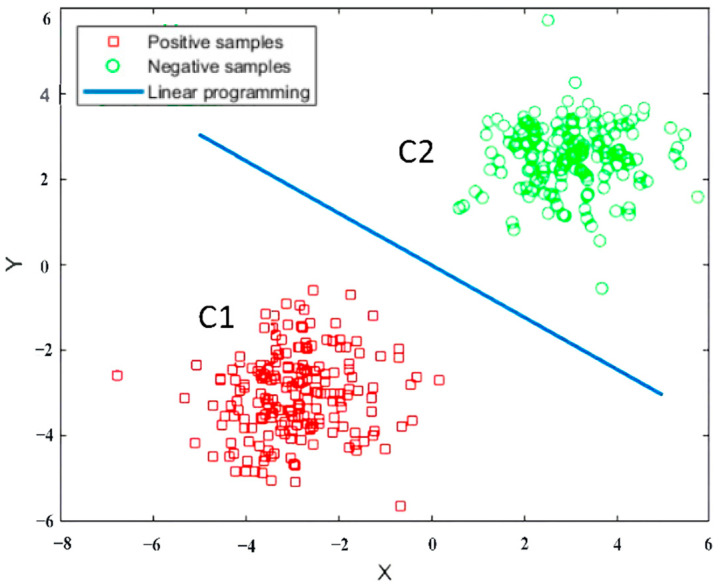
Classification results of linear classifier.

The linear classifier is the simplest and most effective classifier in terms of structure, as shown in Figure 1. For linear functions, there are different forms of existence in different spatial dimensions. Here we have a linear function as follows:

(2) $$g(x)={\;W}^{T}x+b,$$

where *W* denotes the normal vector and *b* is the displacement. $g(x_{i})=0$ indicates the hyperplane (HP). If $g(x_{i})\;>\;0$, it can be judged that $x_{i}$ is C1 class; if $g(x_{i})\;<\;0$, then we can tell that $x_{i}$ is the C2 class. This is equivalent to giving the function *g*(*x*) the symbolic function sign(x), i.e., f(x)=sign[g(x)]:

(3) $$\mathrm{sign}\left[g\left(x\right)\right]=\left\{\begin{array}{c} 1,\;\;x>0\\ 0,\;\;x=0\\ -1,\;\;x<0 \end{array}\right..$$

In binary linear classification, 1 means belonging to this class and −1 means not belonging to this class. Therefore, the margin of a sample point to a certain hyperplane can be defined as follows:

(4) $$\delta_{i\;}={\;y}_{i}(W^{T}x_{i}+b)=\left|g(x_{i})\right|.$$

In the above equation, we put *W* and *b* through the normalization process and *W* and *b* are normalized to $W/\left\|W\right\|$ and $b/\left\|b\right\|$. The geometric interval is

(5) $$\delta_{G\;}=\frac{1}{\left\|W\right\|}\left|g(x_{i})\right|.$$

**Figure 2:**
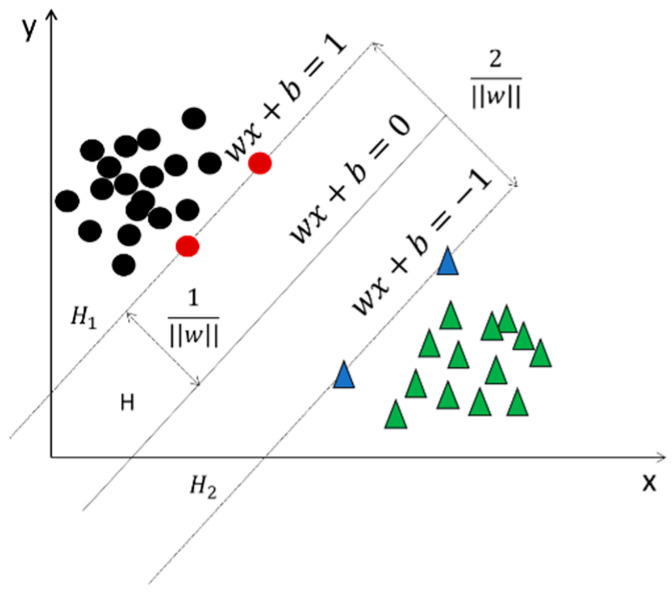
Support Vector Machine.

The result of the above equation is the distance from the point $x_{i}$ in analytic geometry to the line g(x)=0, which is the Euclidean distance from that point to the hyperplane g(x)=0. Figure 2 visualizes the practical meaning of the geometric interval, where *H* is the classification plane, $H_{1}$ and $H_{2}$ are lines parallel to *H* and passing through certain sample points. The distance between *H* and $H_{1}$ ($H_{2}$) is the geometric interval.

### 3.3. Support Vector Machines

The support vector machine (SVM) presents the unique advantages in a variety of applications, such as in solving small sample, nonlinear and high-dimensional pattern sensing [26]. The ultimate goal of the SVM is to identify an optimal hyperplane that separates the sample data, and the principle of separation is to maximize the interval, which is transformed into a convex quadratic programming problem [27]. Depending on whether the training sample can be linearly separated, SVMs are classified into linear SVMs and nonlinear SVMs [28,29].

Given sample data like those in Figure 2, a linear SVM can be implemented if it can be completely and correctly separated by a hyperplane.

Assuming that a minimum value of the interval from the hyperplane is 1 in all sample points, the following equation can be obtained as a constant:

(6) $$y_{i}{{[(W}^{T}x_{i})+b]}^{3}\;\geq \;1\;(i=1,2,\ldots ,l),$$

namely:

(7) $$y_{i}[(W^{T}x_{i})+b]\;-\;1\;\geq \;0\;(i=1,2,\ldots ,l),$$

where l is the total number of samples, $y_{i}$ is 1 for positive samples or −1 for negative samples. The maximization of the geometric interval is transformed into the minimization of ||W||. In addition, in Figure 2, $H_{1}$ and $H_{2}$ pass through the points that are the support vectors, which satisfy the following equation:

(8) $$y_{i}{[(W}^{T}x_{i})+b]=1.$$

From Equation (5), we can obtain the fact that the interval between $H_{1}$ and $H_{2}$ is 2/||W||; the SVM achieves the maximization of the interval, as shown in the following equation:

(9) $$\underset{w,b}{\min}\frac{1}{2}{\left||W|\right|}^{2}\;\mathrm{subject}\;\mathrm{to}\;y_{i}[(W^{T}x_{i})+b]\;-\;1\;\geq \;0\;(i=1,2,\ldots l).$$

The objective function of the above equation is a quadratic function with the linear constraints on W. Such a problem is called quadratic programming (QP). For this type of problem, the Lagrange multiplier can be introduced into Equation (9) by $\alpha_{i}$. The constrained problem is transformed into the optimization problem by constructing a Lagrange function, and the problem is then later converted into solving the Lagrange multiplier after the pairwise α of the problem. This optimization problem is more easily solved. Formally, the equation is as follows:

(10) $$\underset{\alpha }{\min}\frac{1}{2}\sum_{i=1}^{l}\sum_{j=1}^{l}(y_{i}y_{j}x_{i}x_{j}\alpha_{i}\alpha_{j})-\sum_{i=1}^{l}\alpha_{i}\;\mathrm{subject}\;\mathrm{to}\;{\;a}_{i}\;\geq \;0,\forall i,\sum_{i=0}^{N}y_{i}a_{i}=0\;(i=1,2,\ldots l).$$

Therefore, the original solution of W and *b* becomes the solution of α, and thus all unknown parameters of the SVM are solved.

## 4. ECOC-Based SVM Multi-Classification

RFID technology provides clear advantages over other technologies in terms of sensing accuracy, sensing distance, application cost, etc. Moreover, researchers have found that the utilization of RFID backscattered signals can expand the traditional application fields of RFID technology. Based on this discovery, we design and construct an experimental platform for sport motion sensing in this paper based on RFID tags to collect data and use the SVM as the classifier to perform multi-classification through error-correcting output codes (ECOC). Human motion sensing is achieved by constructing multiple binary classification problems.

### 4.1. Principle of ECOC

An ECOC [24] was first applied in the field of communication as a technique to correct information transmission errors during communication. Currently, the ECOC has shown excellent performance in the field of machine learning for solving classification problems of multi-class data.

The ECOC is a method to transform a multi-classification task into multiple binary classification tasks. The ECOC-based SVM multiclassification algorithm can transform a multiclassification problem of K classes into N binary classification problems. When encoding, the ECOC first generates an encoding matrix to train and predict the binary classifier. The encoding matrix is composed of “+1”, “−1” or “0” code values. Each row of the encoding matrix represents a category and each column represents a binary classifier. As shown in Table 1, the classical encoding matrix is a one-to-many coding (ones vs. all, OVA), where B1 to B4 are all binary classifiers, C1 to C4 represent four categories, and the binary classifier only trains the data of the corresponding category with non-zero codes in the column. The training data of the corresponding category of “+1” are considered as a positive category, and the corresponding category of “−1” is considered as a negative category. The first column of the encoding matrix in the following table is [1,−1,−1,−1], i.e., for the binary classifier B1, the data in category C1 are considered as a positive class, and the data in categories C2, C3 and C4 are considered as a negative class. The data in these two categories are used to train the binary classifier B1, and this training method is then applied to the corresponding classifiers in other columns to train the classification.

Notably, three valid conditions need to be satisfied when encoding the ECOC encoding matrix:

(1) The encoding matrix should satisfy the condition that the rows are uncorrelated and non-complementary.

(2) The encoding matrix should satisfy the condition that the columns are uncorrelated and non-complementary.

(3) When classifying class questions, the encoding length should satisfy the condition that $\log_{2}^{k}\;<\;n\;\leq \;2^{k-1}\;-\;1$.

After the encoding matrix is completed, the ECOC trains the binary classifier and all sample data are predicted by each binary classifier to obtain a corresponding result vector. The result vectors are compared with that of each row in the encoding matrix in order, and the ECOC recognizes the corresponding class of the row with higher similarity as the prediction class of the sample. The similarity is obtained by calculating the Hamming distance (HD). The Hamming distance is the number of characters in two equal-length strings that are not the same at the corresponding position. If the vector spacing is small, the similarity is high. If the result vector of a sample is classified as [−1,1,−1,−1], then the hamming distance between the result vector and the corresponding row of C1, C2, C3 and C4 is (2,0,2,2), respectively, and the hamming distance between the result vector and the corresponding row of C2 is the smallest while the sample can be judged to belong to the C2 class.

The ECOC has error correction capability, which can accept partial numbers of binary classifiers in case of classification errors. Its error tolerance is related to the number of binary classifiers. In general, the more binary classifiers there are, the better the error tolerance is. As shown in Table 2, there are three more additional binary classifiers than in Table 1. If there exist data that belong to the C2 class, the prediction result vector obtained in the ideal case that all predictions are correct is [−1,1,−1,−1,−1,1,−1,1]. If the B2 classifier has an error result of −1, the original predicted result vector becomes [−1,−1,−1,−1,1,−1,1], and the Hamming distance between the four categories and the predicted result vector becomes [3,1,3,3], while the category with the minimum decoding distance still belongs to C2, and the classification is still correct. It can be seen that the ECOC encoding matrix can still obtain the correct classification when there is a small amount of classification error.

### 4.2. Encoding Matrix Construction

There are different coding methods depending on the actual classification requirements. The human action sensing in this paper has eight categories, which are coded by random coding. Random encoding includes dense random encoding and sparse random encoding. Dense random coding consists of −1 and +1, and sparse random coding consists of −1, 0 and +1; they therefore belong to binary and ternary coding, respectively. In this paper, the encoding matrix is generated utilizing the dense random coding method consisting of −1 and +1 only. The generation of encoding matrices is implemented programmatically, and for a data set with K categories, the upper limit of the number of possible encoding matrices it can have is $\frac{1}{2}(2^{k}-2)$. For random encoding, the encoding matrix is generated according to the three valid conditions mentioned above, and if an illegitimate encoding column is generated, it needs to be discarded and regenerated until a compliant encoding matrix is generated.

### 4.3. ECOC Decoding

The decoding stage is the last part of the classification, when the result vector is compared with the category codes of each row in the generated encoding matrix, and the corresponding category of the encoding row with greater similarity is taken as the predicted category. Currently, there are binary decode and ternary decode decoding methods. In this paper, the binary decoding method is adopted. The decoding process is based on the distance results obtained from Hamming distance decoding as the content of similarity comparison.

## 5. Performance Evaluation

### 5.1. Experimental Design

In this paper, we investigate indoor sport motion sensing; thus, we select a conference room as the experimental location. The size of the conference room is about $\left(4\times 8\right)m^{2}$. The experimental devices include a laptop computer, an ImpinjR420 reader, a Laird S9028 circularly polarized UHF antenna, 12 Alien9640 passive electronic tags and an antenna fixing bracket.

**Figure 3:**
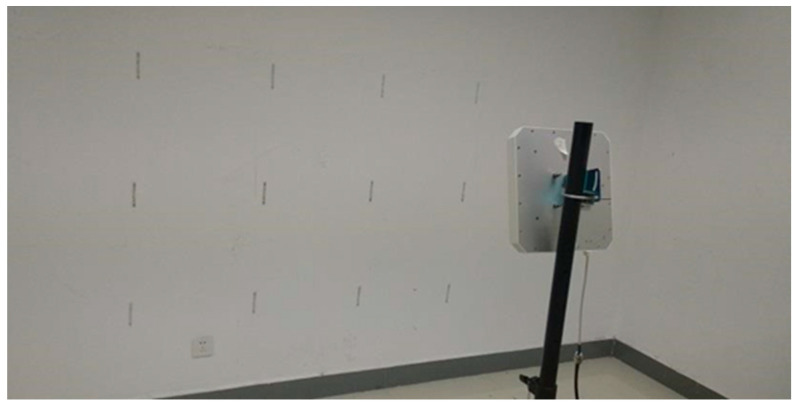
Experimental deployment.

**Figure 4:**
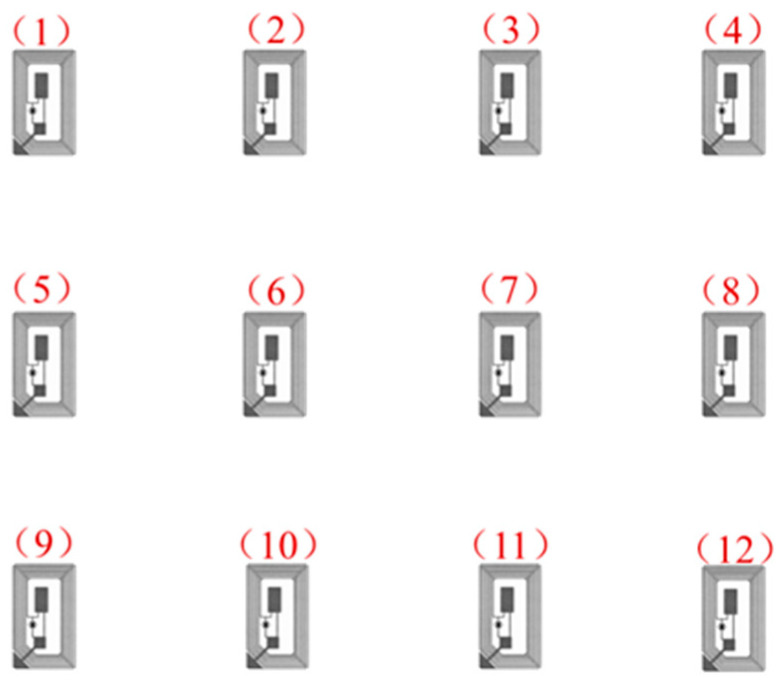
Deployment of electronic tags.

A total of 12 tags is attached to the wall in a 3×4 array, with a spacing distance of 50 cm. The antenna is fixed on the antenna bracket at an angle of 45 degrees facing the wall with the tag array. The distance between the antenna and the wall is 1.38 m, and the antenna is located in the middle of the tag array. The specific experimental deployment is shown in Figure 3 and Figure 4. The human is standing between the antenna and the tags, about 1m away from the antenna, to perform the experimentally set actions.

### 5.2. Experimental Data Presentation

In order to test the generalizability of the proposed method, four volunteers of different heights and weights were invited to perform the experimentally set movements, and Table 3 below shows the height and weight information of the volunteers.

For the corresponding action, each volunteer performs it individually. When different people perform the specified action, there are still slight differences in the process of action execution. Intuitively, the type of action appears to be the same, but it is undeniable that the process of each action execution still preserves the individual micro-actions of the action performer. Take walking as an example: we can observe that although we all walk in our daily lives, the posture of different people is very different. For people around us, we can essentially judge who a person is based on his or her walking posture. Therefore, not interfering with the process of volunteers performing the specified action is also something to pay special attention to during the experiment. If the result classification of different people’s action perception is still accurate in this case, it indicates that the system has high robustness. The experiments in this paper are set up with two types of actions: static poses and dynamic actions. The specific action settings are shown in Table 4.

**Figure 5:**
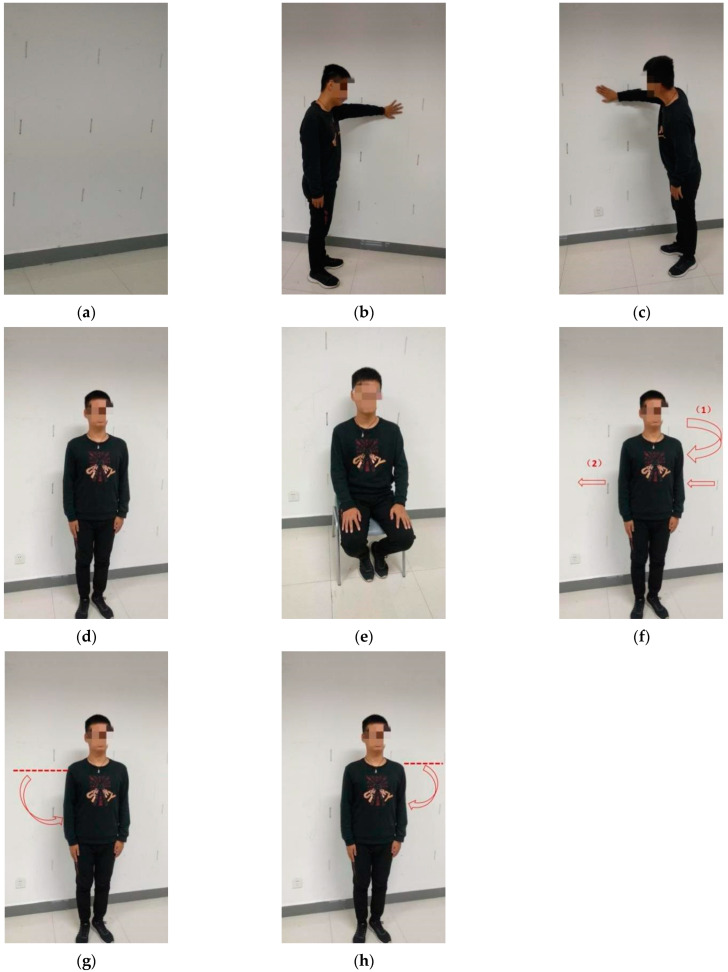
Action acquisition scenes. (**a**) Empty (no one). (**b**) Left hand on the wall. (**c**) Right hand on the wall. (**d**) Standing. (**e**) Sitting. (**f**) Walking. (**g**) Right hand down. (**h**) Left hand down.

Figure 5 shows the action poses performed by one of the volunteers. The volunteer performed the static posture as shown in the figure, where the dynamic action was performed by putting down the right hand horizontally and the left hand horizontally according to the direction of the arrows in the figure, and the volunteer performed the walking action by moving from tabs 4#, 8# and 12# to tabs 1#, 5# and 9# at a normal pace after turning sideways according to the arrow (1).

### 5.3. Feature Parameter Extraction

The data collected are the RSSI values returned to the reader by the electronic tag when the action execution volunteer performs different actions in the experimental scenario. Throughout the data collection process, nearly 60,000 RSSI data were collected for eight action categories: unoccupied (empty), left hand on wall, right hand on wall, standing, sitting, walking, right hand from horizontal down and left hand from horizontal down. All static pose data were collected for 10 s, with a sampling rate of about 5 per second, for a total of about 50 pieces of data as a set of samples. The dynamic action part imitates the action in the real environment, and the duration of each action is about 4 s; therefore, each dynamic action is collected for about 20 RSSI values. The specific number of samples for different action types is shown in Table 5.

**Figure 6:**
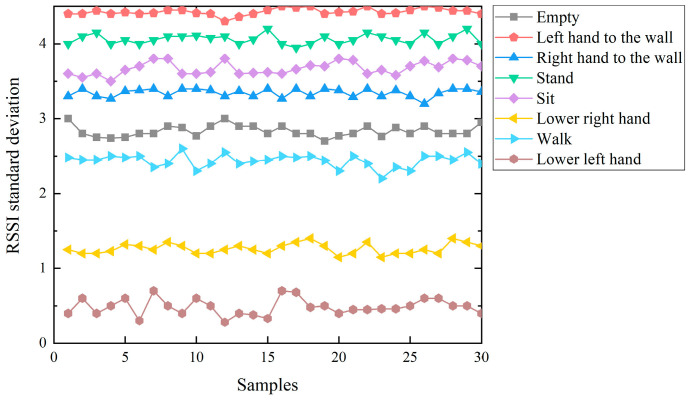
Standard deviation of different actions.

During the process of gathering data pertinent to static poses and subsequent data analysis, it was observed that the RSSI values emitted by the 12 tags exhibited a notable degree of stability. The maximal deviation between the upper and lower bounds of these values did not exceed 1. Consequently, when extracting feature values, the RSSI values from the 12 tags were amalgamated into a singular group for subsequent extraction. The resulting feature values encompassed the maximum, minimum, mean, and standard deviation values. Conversely, for dynamic actions, feature values were derived from each tag individually, capturing their respective maximum, minimum, mean, and standard deviation values. The variation in the RSSI standard deviation across eight distinct action types is depicted in Figure 6. It is discernible that different actions exhibit distinct trends in standard deviation, underscoring the significance of this metric as a distinguishing feature. The capacity of varying actions to be delineated from one another through standard deviation lends credence to its viability as a feature for discerning diverse sport motion actions.

### 5.4. Analysis of Experimental Perception Results

**Figure 7:**
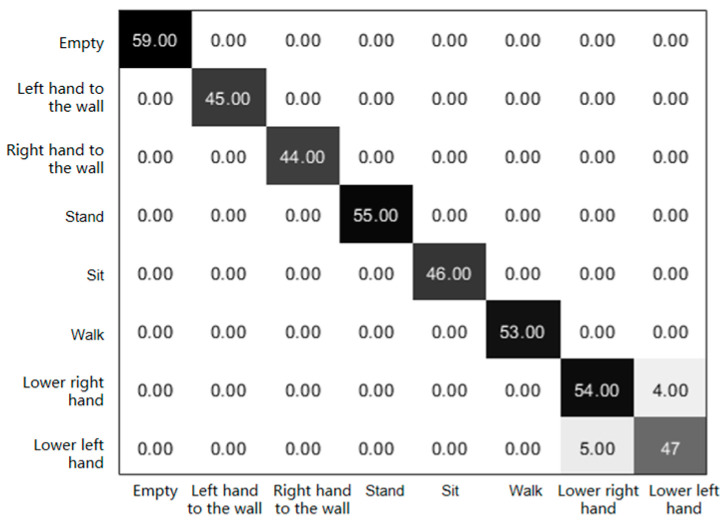
Confusion matrix of different sport motion sensing results.

**Figure 8:**
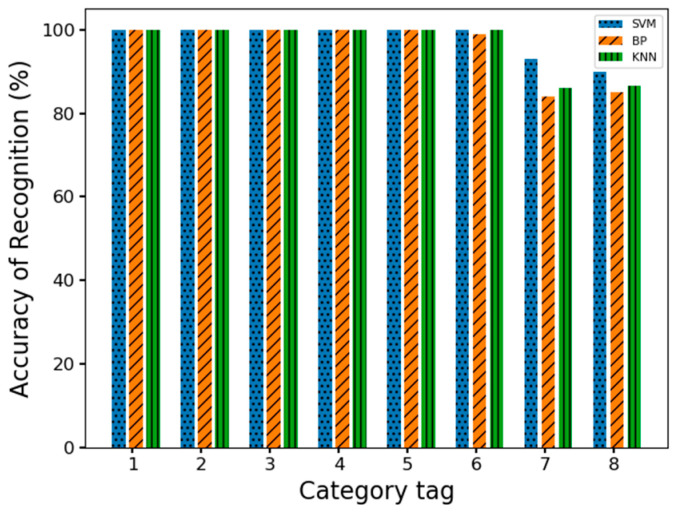
Comparison of accuracy of three classification methods.

After extracting the feature values, we then used them as training samples for sport motion sensing classification using the previously introduced ECOC-SVM multi-classification method. In this case, the training sample data account for 75% of the total sample data and the test data are 25%. Figure 7 portrays the confusion matrix that encapsulates the diverse accuracies achieved through the application of this classification approach in sport motion sensing. Meanwhile, Figure 8 visually presents the outcomes of the classification process, delineating the classification results for the eight distinct actions across eight distinct labeling categories.

Figure 7 illustrates the accuracy graph depicting the classification outcomes attained through the methodology. The vertical axis corresponds to the actual categorization of actions, while the horizontal axis corresponds to the predicted action classifications. Taking the instance of the “standing” action as an illustrative example, the test dataset comprises 55 true samples for the “standing” motion. The predicted outcome aligns with the value 55 within the depicted black block, indicating a classification accuracy of 100% for this specific action. If we observe the graphical representation, it becomes evident that the classification accuracy for the five static motions, namely “empty” (absence of motion), “left hand on the wall”, “right hand on the wall”, “standing”, and “sitting”, is uniformly 100%. Furthermore, within the realm of dynamic actions, the accuracy for sensing the actions of “walking”, “right hand down”, and “left hand down” attains values of 100%, 93.1%, and 90.4%, respectively.

At the same time, the data collected from this experimental environment were subjected to the same data processing to extract the maximum, minimum, mean, and standard deviation of the same feature values, and two classical classification algorithms, the KNN (k-Nearest Neighbor) and BP neural network, were selected to recognize and classify the above eight different actions. For the KNN, we cross-validate by grid search, starting with a smaller *k* value and increasing the *k* value, and then calculating the variance of the verification set to finally find a more suitable *k* value. For the BP neural network, we utilize the cross-entropy loss between the outputs and the true labels as the loss function and select 1 as the hyperparameter. From Figure 8, it can be seen that all three algorithms achieved better results in the sensing and classification of static motions, but in the sensing and classification of dynamic motions the sensing and classification accuracy of the SVM is greater compared to the other two algorithms. Moreover, Table 6 shows the results regarding the precision, recall and F_1_-score of our proposed method, which can further demonstrate the performance advantage of the method.

## 6. Conclusions

In this paper, we propose an RFID-based sport motion sensing system, which uses an SVM as a binary classifier and combines an ECOC multi-classification strategy to classify eight different actions. The system is human-friendly, is non-invasive and does not require the human body to wear a sensor. It can achieve high sensing accuracy for a variety of human sport motions and can be applied to scenarios such as homes for the elderly, kindergartens, and hospitals to achieve better human supervision, with great application value and broad application prospects. However, the system’s accuracy may be affected by factors such as environmental interference and RFID tag placements. Additionally, its application may be constrained in scenarios where highly precise motion classification is essential. Thus, future research should concentrate on enhancing the system’s robustness against environmental influences, devising adaptive algorithms to accommodate individual differences, and exploring methods to expand its application scope. Moreover, investigating real-time tracking capabilities and optimizing hardware components could further elevate the system’s utility in dynamic environments. These directions could collectively bolster the system’s accuracy, versatility, and practical applicability, contributing to the advancement of human motion sensing technology.

## Figures and Tables

**Table 1:** Ones vs. all coding.

Category	Code			
	B1	B2	B3	B4
C1	1	−1	−1	−1
C2	−1	1	−1	−1
C3	−1	−1	1	−1
C4	−1	−1	−1	1

**Table 2:** OVA growth matrix.

Category	Code						
	B1	B2	B3	B4	B5	B6	B7
C1	1	−1	−1	−1	1	1	−1
C2	−1	1	−1	−1	1	−1	1
C3	−1	−1	1	−1	−1	1	1
C4	−1	−1	−1	1	−1	−1	−1

**Table 3:** Information for volunteers.

Serial Number	Height	Weight
Volunteer A	175 cm	67 kg
Volunteer B	171 cm	65 kg
Volunteer C	179 cm	75 kg
Volunteer D	174 cm	65 kg

**Table 4:** Type of action.

Action Type	
Static Posture	No one (empty)
	Left hand on the wall
	Right hand on the wall
	Standing
	Sitting
Dynamic Action	Walking
	Right hand down
	Left hand down

**Table 5:** Number of action samples collected.

Action Type	
No one (empty)	236
Left hand on the wall	180
Right hand on the wall	176
Standing	220
Sitting	184
Walking	212
Right hand down from horizontal	232
Left hand down from horizontal	208

**Table 6:** Precision, Recall and F_1_-score of our proposed method.

Classification Results	Precision (%)	Recall	F_1_-Score
1	100	1	1
2	100	1	1
3	100	1	1
4	100	1	1
5	100	1	1
6	100	1	1
7	93.1	91.5	92.3
8	90.3	92.2	91.2

## Data Availability

The data are not publicly available due to the privacy of individuals involved.
